# Clinical characteristics of enteroviral meningitis without pleocytosis in children: a retrospective single center observational study in the Republic of Korea

**DOI:** 10.1186/s12887-019-1714-1

**Published:** 2019-09-14

**Authors:** Yura Ko, Woochan Jeon, Minjung Kathy Chae, Heewon Yang, Jisook Lee

**Affiliations:** 1 Department of Emergency Medicine, Ajou University School of Medicine, Suwon, Republic of Korea; 2 Department of Emergency Medicine, Inje University, Ilsan Paik Hospital, Goyang, Republic of Korea

**Keywords:** Enterovirus, Meningitis, Pleocytosis, Child, RT-PCR

## Abstract

**Background:**

We aimed to study the prevalence of enterovirus (EV) meningitis without the presence of cerebrospinal fluid (CSF) pleocytosis and identify patient factors and clinical features associated with it.

**Methods:**

This was a retrospective analysis of patients aged < 18 years old who were diagnosed with EV meningitis by CSF reverse-transcriptase polymerase chain reaction (RT-PCR) testing between January 2015 and December 2016. Clinical variables were compared with regard to the presence of CSF pleocytosis.

**Results:**

A total of 305 patients were enrolled in study; 169 (55.4%) had no pleocytosis. Patients without pleocytosis were younger (median age 2 months vs. 67.0 months, *p* < 0.01) and had lower white blood cell (WBC) count (median, 8600/mm^3^ vs. 10,300/mm^3^, *p* < 0.01). Also absolute neutrophil (ANC) count were lower than pleocytosis group (median, 4674/mm^3^ vs. 7600/mm^3^, *p* < 0.01). Comparing three age groups, CSF apleocytosis was present in 106 of 128 patients (82.8%) aged ≤3 months, 7 of 13 patients (53.8%) aged 3 months–3 years and 56 of 164 patients (34.1%) aged > 3 years. Younger age groups had higher prevalence of CSF apleocytosis (*p* < 0.01). In patients aged ≤3 months, 94.5% underwent lumbar puncture within 24 h of symptom onset. The frequency of not having pleocytosis was higher than the frequency of having pleocytosis during peak EV infection prevalent months (summer and fall) (*p* < 0.01).

**Conclusion:**

This study shows that EV meningitis in young infants, with early lumbar puncture, or occurring during peak EV meningitis prevalent seasons cannot be solely excluded by pleocytosis. Also, a confirmation test for EV meningitis should be performed using RT-PCR.

## Background

Viral meningitis is characterized by the acute onset of fever, headache, and neck stiffness, often accompanied by nausea and vomiting. Non-polio enteroviruses (EV), which are a genus in the family Picornaviridrae, are known as the most common pathogen of aseptic meningitis in children [1] and account for approximately 60–80% of aseptic meningitis cases [1–3]. The outcomes of EV infections of the central nervous system are usually favorable in otherwise healthy patients, but EV meningitis causes considerable morbidity, with moderate or high fever despite antipyretics and several days of severe headache warranting opiate analgesia [1, 4]. Thus, careful attention is needed to confirm the diagnosis.

Several studies have reported the lack of pleocytosis in children with EV meningitis, RT-PCR is presently considered to be the gold standard for diagnosis of EV infections [2]. CSF analysis takes several hours, but RT-PCR requires several days depending on the facilities; therefore, it is difficult to confirm EV via RT-PCR during a patient’s stay in the emergency department (ED). Although disposition should be based on suspicion of meningitis and symptom severity rather than the presence of CSF pleocytosis, in reality, due to lack of medical resources many patients are discharged from the ED if CSF cell count is normal with well controlled symptoms. Some of these patients revisited the ED with aggravated symptoms, upon which EV meningitis was confirmed via positive CSF RT-PCR results using records of the previous ED visit. Thus, we aimed to analyze the prevalence of enterovirus (EV) meningitis without the presence of cerebrospinal fluid (CSF) pleocytosis and identify patient factors and clinical features.

## Materials and methods

### Patients

We retrospectively reviewed medical records of patients < 18 years old who visited the pediatric ED of Ajou University Hospital, South Korea, which is a tertiary referral center. We enrolled the patients who underwent CSF analysis and CSF EV RT-PCR testing in the ED between January 2015 and December 2016. All patients with positive CSF RT-PCR, negative blood and CSF bacterial cultures were finally diagnosed as EV meningitis and included in this study. Patients who did not fulfill the criteria or had incomplete medical records were excluded. Ethical approval was obtained from local institutional review board.

### Study definitions

CSF pleocytosis was defined as CSF white blood cell (WBC) count > 22 cells/mm^3^ for age < 4 weeks, > 15 cells/mm^3^ for age 4–8 weeks, and > 5 cells/mm^3^ for age > 8 weeks [5]. In a traumatic lumbar puncture, the pleocytosis was corrected as the proportion of WBCs to red blood cells (RBC) in the spinal fluid is 1:500 [6, 7].

### EV RT-PCR

The AccuPower EV real-time RT-PCR kit (Bioneer, Republic of Korea) was used for the detection of EV ribonucleic acid (RNA) through RT-PCR amplification of the 5′-untranslated region of the EV genome. Nucleic acid extraction was performed using ExiPrep™ 16 Dx, extracted RNA was combined with the Enterovirus PCR premix, and the Exicylcer™96 Real-Time Quantitative thermal block was used for detection of the amplified product. This kit detects 52 enterovirus serotypes. The entire process takes 2–3 days before obtaining results of RT-PCR.

### Data collection and statistical methods

Data collected included demographics, chief complaint of visit, symptoms, time interval from symptom onset of meningitis to timing of lumbar puncture, hospitalization, and ED re-visit rate. We also collected signs of meningeal irritation such as neck stiffness and nuchal rigidity (Kernig sign and Brudzinski sign) [8] from retrospective chart review. Laboratory results of blood, CSF chemistry and CSF virological test were obtained from the electronic medical records.

Clinical and laboratory variables were compared between the two groups according to the presence of CSF pleocytosis. Continuous variables are described as the median with interquartile range (IQR) or mean ± standard deviation. Categorical variables are presented as the frequency or percentage. Chi-squared test or the Fisher’s exact test was used for the analysis of categorical data and the Student’s t test for continuous variables with normal distribution. Mann–Whitney test was used for continuous variables without a normal distribution. Statistical analyses were performed using SPSS 15.0(Windows Inc., Chicago, IL, USA). *p* values less than 0.05 were considered statistically significant.

## Results

### Characteristics of patients diagnosed with EV meningitis

A total of 305 patients were enrolled in the study. Median age of analyzed patients was 47.0 months (IQR 1.0–78.0); 189 (62%) were male. Fever was the most common symptom (96.7%), followed by headache (51.8%), vomiting (43.9%), and seizure (1.6%). Meningeal irritation sign was positive in 90 patients (29.5%). Patient characteristics and laboratory results are shown in Table 1.

**Table 1:** Characteristics of patients diagnosed with enteroviral meningitis

	Patients diagnosed with enteroviral meningitis (*N* = 305)
Clinical characteristics	
Age, months^a^	47.0 (1.0–78.0)
Male, n (%)	189 (62.0%)
Fever, n (%)	295 (96.7%)
Headache, n (%)	158 (51.8%)
Vomiting, n (%)	134 (43.9%)
Seizure, n (%)	5 (1.6%)
Meningeal irritation, n (%)	
Negative	197 (64.6%)
Equivocal	18 (5.9%)
Positive	90 (29.5%)
Laboratory characteristics	
Peripheral WBC (/mm3)^a^	9500 (6900 - 11,950)
ANC (/mm3)^a^	5589 (3375.8 – 88,804.0)
CRP (mg/dl)^a^	0.7 (0.3–1.2)
CSF WBC (/mm3)^a^	5 (2–40)
CSF ANC (/mm3)^a^	0.0 (0.0–11.4)
CSF RBC (/mm3)^a^	2 (0.0–15.8)
CSF protein (mg/dl)^a^	37.0 (26.0–53.9)
CSF/Serum glucose ratio^a^	0.6 (0.5–0.6)

*WBC* white blood cell, *ANC* absolute neutrophil Count, *CRP* c-reactive protein, *CSF* cerebrospinal fluid, *RBC* red blood cell

^a^ median (Interquartile range)

### Clinical and laboratory differences between patients with and without pleocytosis

Table 2 shows the clinical differences between patients with and without pleocytosis. Patients without pleocytosis were younger than patients with pleocytosis (median, 2.0 months vs. 67.0 months, *p* < 0.01). Fever was common in both groups. However, vomiting (62.5%) was more frequent in patients with pleocytosis. Headache was more frequently present in children older than 3 years of age possibly due to the ability to communicate symptoms. None of the infants younger than 3 months of age had documented headache. Only 4 of 13 preverbal aged children (from 3 months old to 3 years old) reported headache; 2 patients had pleocytosis and 2 patients did not have pleocytosis. In 164 patients older than 3 years, patients complained of headache regardless of pleocytosis; 103 with pleocytosis vs. 51 without pleocytosis..

**Table 2:** Clinical differences between patients with pleocytosis and patients without pleocytosis

	With pleocytosis (*n* = 136)	Without pleocytosis (*n* = 169)	*P*
Age (months) ^a^	67.0 (43.3–87.8)	2.0 (1.0–63.5)	0.00
Male, n (%)	86 (63.2)	103 (60.9)	0.68
Fever, n (%)	131 (96.3)	164 (97.0)	0.76
Vomiting, n (%)	85 (62.5)	19 (29.0)	0.00
Seizure, n (%)	2 (1.5)	3 (1.8)	0.84

^a^ median (Interquartile range)

Regarding laboratory differences, patients without pleocytosis showed lower WBC (*p* < 0.01) and ANC count (*p* < 0.01). Though patients with pleocytosis showed a higher serum/CSF glucose ratio (median, 0.6 vs. 0.6, *p* = 0.01) and lower CSF protein levels (median, 35.0 mg/dl vs. 40.6 mg/dl, *p* = 0.06) than patients without pleocytosis; the values of both groups were within the normal range (Table 3).

**Table 3:** Laboratory differences between patients with pleocytosis and patients without pleocytosis

	With pleocytosis (*n* = 136)	Without pleocytosis (*n* = 169)	*P*
Peripheral WBC (/mm3) ^a^	10,300 (8200 - 13,000)	8600 (6000 - 10,700)	0.00
ANC (/mm3) ^a^	7600 (4447.5 - 10,252.5)	4674 (2988.5 - 7596.3)	0.00
CRP (mg/dl) ^a^	0.6 (0.3–1.2)	0.7 (0.3–1.3)	0.65
CSF WBC (/mm3) ^a^	43 (19–110)	2 (1–4)	0.00
CSF RBC (/mm3) ^a^	2 (0–11)	1 (− 36)	0.30
CSF protein (mg/dl) ^a^	35 (24.9–46.2)	40.6 (26.7–61.1)	0.06
CSF/Serum glucose ratio^a^	0.6 (0.5–0.7)	0.6 (0.5–0.6)	0.01

*WBC* white blood cell, *ANC* absolute neutrophil Count, *CRP* c-reactive protein, *CSF* cerebrospinal fluid

^a^median (Interquartile range)

### Clinical differences by age group and frequency of apleocytosis according to month of year

When analyzing clinical differences according to age group, CSF apleocytosis was present in 106 patients (82.8%) aged ≤3 months, 7 patients (53.8%) aged 3 months - 3 years, and 56 patients (34.1%) aged > 3 years; CSF apleocytosis was more common in younger groups (*p* < 0.01; Table 4). While fever was the most common presentation in patients aged ≤3 months (99.2%), patients aged > 3 years had more frequent clinical signs and symptoms suspicious for meningitis such as fever (94.5%), headache (93.9%), and vomiting (78.7%). On the other hand, a lumbar puncture was performed within 24 h of symptom onset in 94.5% of patients aged ≤3 months, whereas it was performed within 24 h of symptom onset in 57.3% of patients aged > 3 years.

**Table 4:** Clinical differences among the age groups

Age group	≤ 3 months (*n* = 128)	3 months - 3 years (*n* = 13)	> 3 years (*n* = 164)	*P*
Apleocytosis, n (%)	106 (82.8)	7 (53.8)	56 (34.1)	0.00
Fever, n (%)	127 (99.2)	13 (100)	155 (94.5)	0.10
Headache, n (%)	0 (0)	4 (30.8)	154 (93.9)	0.00
Vomiting, n (%)	1 (0.8)	4 (30.8)	129 (78.7)	0.00
Seizure, n (%)	0 (0)	3 (23.1)	2 (1.2)	0.00
Less than 24 h from symptom to Lumbar puncture, n (%)	121 (94.5)	7 (53.8)	70 (42.7)	0.00

**Fig. 1:**
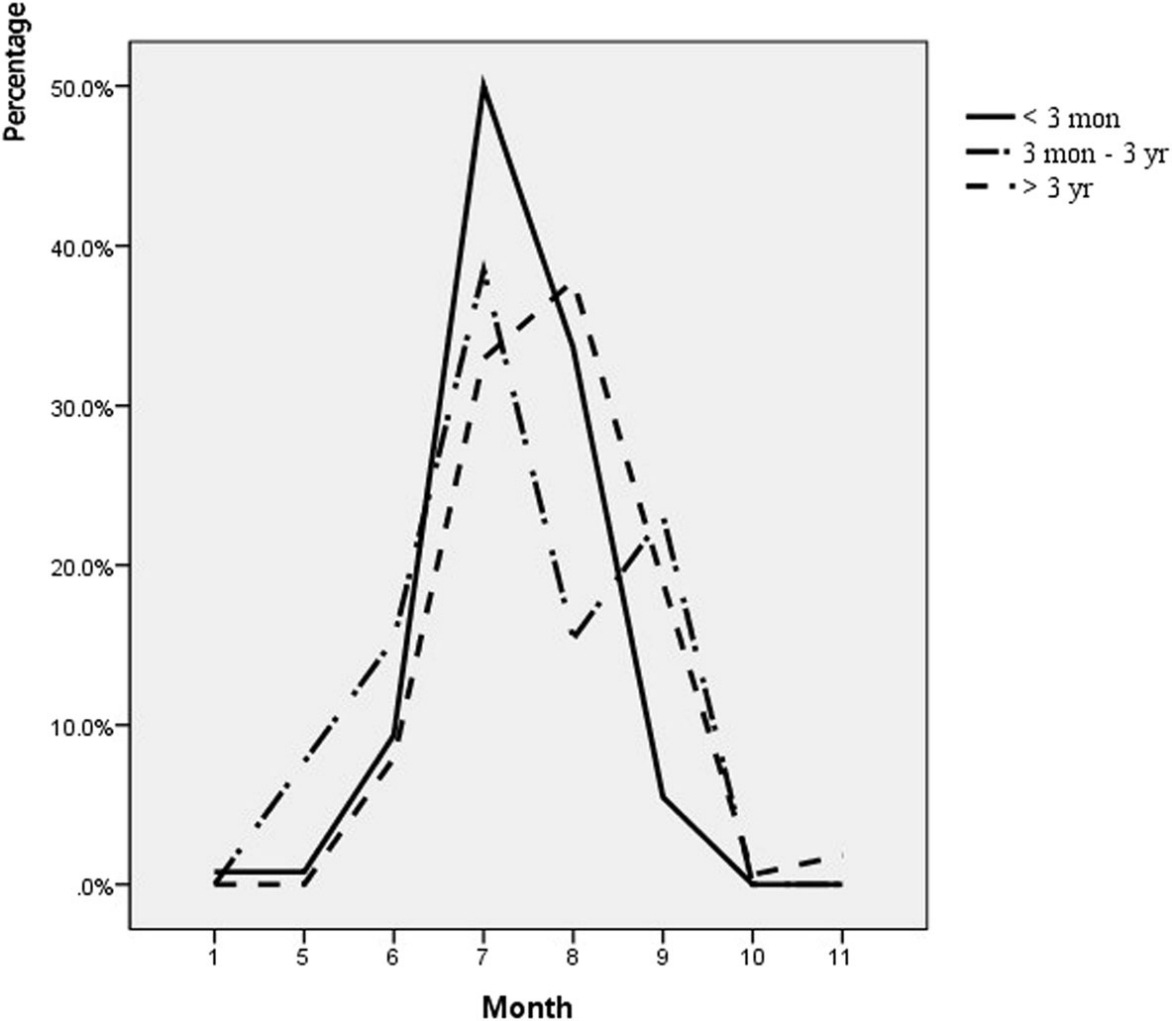
Annual Visits in ED according to age groups. During summer and early fall, visits in emergency department were peaked among all age groups

**Fig. 2:**
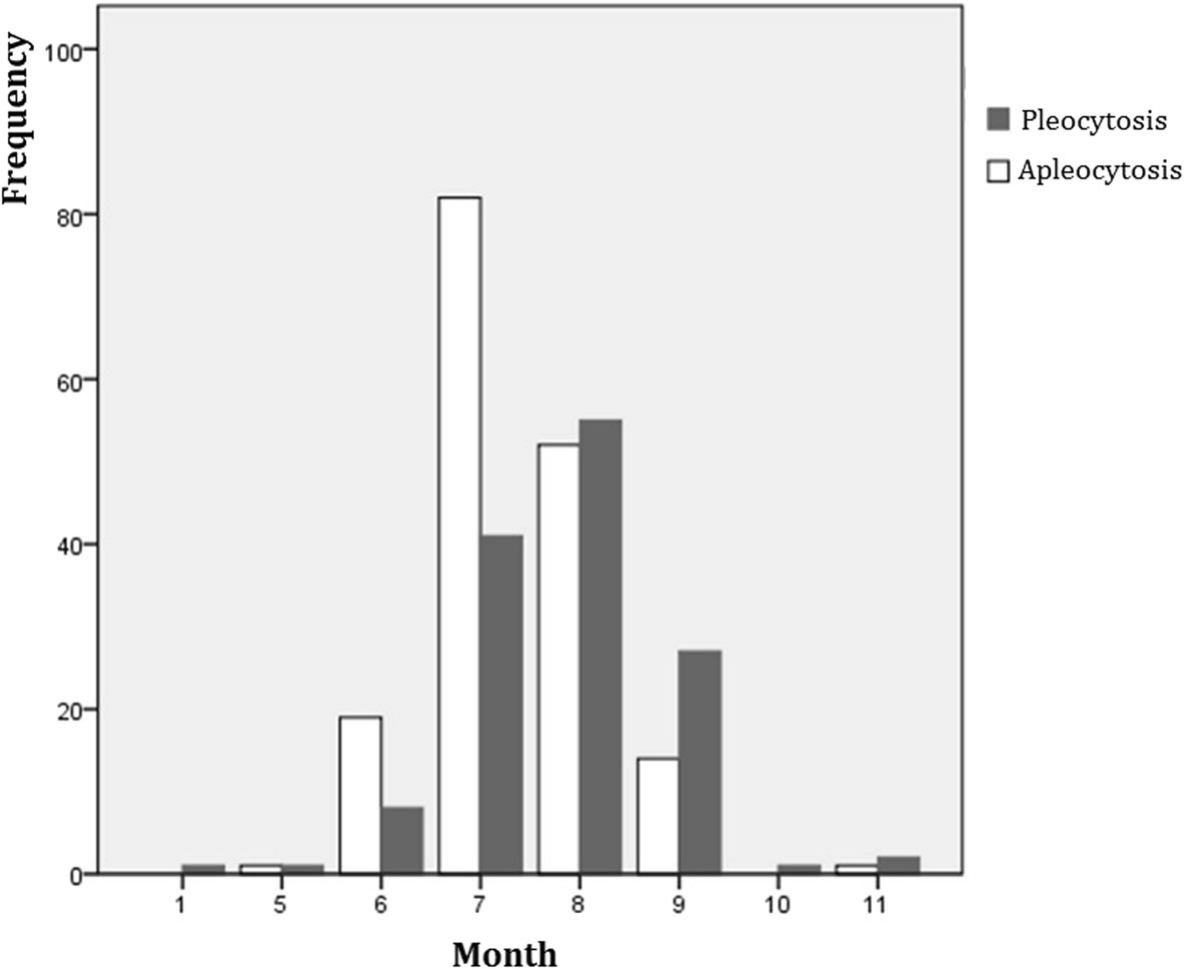
The frequency of apleocytosis according to month of the year. During June and July, which are periods of peak EV prevalence, the frequency of not having pleocytosis was higher than the frequency of having pleocytosis

Figures 1 and 2 shows the frequency of pleocytosis according to month of the year. During June and July which are periods of peak EV prevalence [2, 9, 10], the frequency of not having pleocytosis was higher than the frequency of having pleocytosis (*p* < 0.01).

## Discussion

We studied 305 cases of RT-PCR-confirmed EV meningitis—the largest number of cases from a single-center study—and compared clinical features of patients and the frequency of pleocytosis during periods of EV prevalence.

This study shows that patients with apleocytosis were younger, had lower WBC and ANC count, compared with those characteristics in patients with pleocytosis. Several studies have reported the absence of CSF pleocytosis in children with EV meningitis [7, 11–14]. For example, Yun et al. reported that about 30% of patients aged < 3 months with EV meningitis did not have CSF pleocytosis [7]. Other studies report a similar proportion of cases lacking pleocytosis in EV meningitis among patients aged < 3 months: 34% of patients aged < 90 days in Singapore [12], 31% in Pennsylvania, US [13], and 30% of patients aged < 2 months in Missouri, US [15]. Furthermore, a study from the Netherlands also showed that 40% of pediatric patients aged < 16 years with EV meningitis did not demonstrate CSF pleocytosis and these patients were significantly younger (*p* = 0.001) [11]. Compared with previous studies, we showed higher prevalence of EV meningitis with apleocytosis; overall 55.4, and 82.8% in patients younger than 3 months.

The mechanism underlying the lack of pleocytosis in younger patients has been suggested to be connected with the immaturity of the immune system of young patients [13]. Mulford et al. speculated that the immunologic maturity of these patients is insufficient to recruit leukocytes to the site of infection [15]. Moreover, consistent with the current results, lower peripheral WBC count in patients without pleocytosis has also been observed in other studies [7, 11, 13]. The association between lower peripheral WBC count and a lack of CSF pleocytosis in young patients may also be explained by immunologic immaturity [11, 13].

Another hypothesis to explain the frequent absence of pleocytosis at younger ages is the short time from the onset of EV meningitis to lumbar puncture [7, 12]. Yun et al. hypothesized that the immunologic response that recruits WBCs to CSF may not be complete due to the short duration of symptoms [7]. In the present study, 82.8% of infants younger than 3 months had a higher frequency of the absence of pleocytosis than that in previous studies. The reason for this higher rate might be because 94.5% of patients aged ≤3 months underwent lumbar puncture within 24 h. In Korea, domestic caregivers are taught that fever in infants is a dangerous symptom of infection, and they can easily access an emergency room at any time [16]. Especially if the child is younger, caregivers visit the outpatient clinic or emergency room as soon as possible, so the examination and diagnosis tends to be done quickly [17].

Heo et al. reported a lower CSF protein level in patients without pleocytosis (apleocytosis group 18.9 ± 4.3 mg/dl vs. pleocytosis group 34.9 ± 18.3 mg/dl, *p* = 0.002) and explained that at the initial stage of illness, blood-brain barrier permeability is maintained, thus WBCs have not yet infiltrated the cerebrospinal cavities and CSF protein level dose not increase [18]. On the other hand, the CSF protein level in patients without pleocytosis was higher in our study, and this is in discord with another study [14]. To explain our result, we must consider the normal protein level in CSF according to age. The normal range of CSF protein level according to the actual age is higher in neonates and infants than it is in children (preterm infant 115 mg/dl (65–150), term neonate 90 mg/dl (20–170), child 5-40 mg/dl) [19, 20]. Our result suggests that the higher protein level in patients without pleocytosis is because the apleocytosis group contained more patients aged ≤3 months.

Additionally, our results reveal that patients with pleocytosis had complain of more meningitis symptoms such as headache and vomiting than patients without pleocytosis. This might be because those with pleocytosis were older, had more advanced verbal skills, and thus could verbally explain their symptoms such as headache more easily.

Another important finding from our analysis is that there was a significantly different frequency of pleocytosis according to season of the year. EV meningitis shows a pronounced seasonality, being more frequent in summer and fall [9, 10]. In our study, while the frequency of EV meningitis without pleocytosis was higher compared with pleocytosis meningitis during prevalent EV seasons, the frequency was similar during non-predominant months. During the traditional EV season, we recommend that patients with symptoms suspicious for meningitis undergo a lumbar puncture, with RT-PCR testing.

The diagnosis of meningitis affected the dispositions; discharge or admission. Among 305 patients, 43 patients (14.1%) were discharged from the ED. In patients with pleocytosis, all were hospitalized except for one; the remaining discharged 42 patients did not demonstrate pleocytosis. 6 patients of these 42 patients (14.3%) revisited the ED with aggravated symptoms and poor condition. When they returned to the ED, the results of RT-PCR during the previous visit were confirmed, and they finally received a diagnosis of EV meningitis without pleocytosis. This suggests that although the first result of the CSF profile was negative, patients with clinical features suspicious for meningitis should have their diagnosis confirmed by RT-RCR.

This study has several limitations. First, because of the retrospective design, clinical features such as vomiting, headache, irritability and meningeal irritation sign may be inadequate based solely on medical records. Also, as younger patients may not have been able to communicate their symptoms, this data in the younger age group may not be accurate. As retrospective medical reviews, we had small numbers in 3 months - 3 years. Though we analyzed non-parametric methods, this is weak point of our study. Second, this study was a single-center study, thus the results may not be widely applicable. In addition, our RT-PCR data contained only positive or negative results of EV infection and did not indicate a subtype; accordingly, we were unable to determine whether differences in the present study and those of previous reports are due to EV subgroup differences. For example, compared with previous studies, we found a higher frequency of apleocytosis in older children, but this can be influenced by EV subtype. Taken together, we consider that a prospective multicenter study is needed to address these points.

## Conclusion

We confirmed that EV meningitis without pleocytosis can occur commonly. EV meningitis in young infants, with early lumbar puncture, or during the prevalent EV infection season cannot be solely excluded by the absence of pleocytosis; such cases should undergo timely confirmation testing using RT-RCR.

## Data Availability

The study data is available from the corresponding author on reasonable request.

## Abbreviations

ANC: Absolute neutrophil count
CFS: Cerebrospinal fluid
ED: Emergency department
EV: Enterovirus
RT-PCR: Reverse-transcriptase polymerase chain reaction
SD: Standard deviation
WBC: White blood cell
